# dnoise: Fast Native Data Reduction for Bruker timsTOF

**DOI:** 10.64898/2026.08.27.747603

**Published:** 2026-09-01

**Authors:** Patrick T. Garrett, Jolene K. Diedrich, John R. Yates

## Abstract

Bruker timsTOF acquisitions produce dense native .d files whose storage, transfer, and archival become substantial at high throughput. We present dnoise, an open-source Rust tool that removes points directly from timsTOF frames and writes a native-compatible .d directory. dnoise retains ions that form coherent streaks across the ion-mobility dimension and applies acquisition-aware gates to signal that cannot be selected for fragmentation. On a three-species benchmark spanning ddaPASEF and diaPASEF at 5- and 15-minute gradients, default MS1-only denoising reduced the frame binary by 35 to 53%. Label-free quantification accuracy was preserved in both modes. ddaPASEF peptide-spectrum-match, peptide, and protein-group counts were unchanged, as expected with the searched MS/MS spectra untouched, and diaPASEF precursor and protein-group counts changed only slightly. Every tested processing run completed in 69 seconds or less on the benchmark workstation. Optional MS/MS denoising produced greater reduction but sacrificed several percent of identifications. Thus, a substantial fraction of native timsTOF frame data can be removed with little analytical change.

**Abstract Figure:**

## Full Text Availability

The license terms selected by the author(s) for this preprint version do not permit archiving in PMC. The full text is available from the preprint server.

